# Impact of an eConsult-based antimicrobial stewardship program using the ExpertTWIN system: a Bayesian structural time-series analysis

**DOI:** 10.1017/ash.2026.10851

**Published:** 2026-09-07

**Authors:** Naoya Itoh, Tomoyo Mizuno, Chiharu Wachino, Shunsuke Kuriki, Masaya Nagamizu, Nana Akazawa-Kai, Nobumasa Okumura, Kento Furuya, Kaku Yu, Takanori Kawabata

**Affiliations:** 1 Department of Infectious Diseases, Graduate School of Medical Sciences, Nagoya City University, Aichi, Japan; 2 Department of Clinical Infectious Diseases, Graduate School of Medical Sciences, Nagoya City University, Aichi, Japan; 3 Department of Infectious Diseases, Nagoya City University East Medical Center, Aichi, Japan; 4 Department of Pharmacy, Nagoya City University West Medical Center, Aichi, Japan; 5 Department of Clinical Pharmaceutics, Graduate School of Medical Sciences, Nagoya City University, Aichi, Japan; 6 Department of Pharmacy, Nagoya City University East Medical Center, Aichi, Japan; 7 Department of Data Science, National Cerebral and Cardiovascular Center, Osaka, Japan

## Abstract

**Objective:**

Antimicrobial stewardship programs (ASPs) are difficult to implement in hospitals with limited access to infectious disease (ID) specialists. Electronic consultation–based tele-stewardship may offer a scalable solution; however, evidence regarding its implementation and impact remains limited. This study evaluated the impact of an eConsult-based remote antimicrobial stewardship program on antimicrobial consumption and resistance patterns.

**Design:**

Single-center retrospective observational study.

**Setting:**

A 500-bed tertiary care hospital without on-site ID specialists.

**Patients:**

In total, 27,863 patients were hospitalized.

**Interventions:**

An asynchronous eConsult-based tele-stewardship program using the ExpertTWIN system was implemented in February 2025. Data from February 2024 to January 2026 were analyzed using a Bayesian structural time-series model.

**Results:**

During the intervention period, 145 cases were reviewed. Carbapenem use decreased; use of antipseudomonal and narrow-spectrum agents showed evidence of increases, whereas use of anti-MRSA agents and total oral and intravenous antimicrobial use showed evidence of reductions. The incidences of carbapenem-resistant *Pseudomonas aeruginosa*, two-drug-resistant *P. aeruginosa*, and MRSA showed evidence of reductions, whereas third-generation cephalosporin-resistant *Escherichia coli* increased. Culture specimen submission showed evidence of an increase. There was no clear evidence of reductions in all-cause in-hospital mortality or length of stay. Total antimicrobial costs were unchanged.

**Conclusions:**

The eConsult-based tele-stewardship program reduced carbapenem use and was associated with favorable changes in several antimicrobial-use, resistance, and diagnostic measures, although third-generation cephalosporin-resistant *E. coli* increased. No clear changes in clinical outcomes were observed. This model may offer a practical and scalable ASP strategy for hospitals without on-site ID specialists.

## Introduction

Antimicrobial stewardship programs (ASPs) are essential interventions designed to optimize antimicrobial use, reduce antimicrobial resistance, and improve patient outcomes.^1^ However, implementing effective programs remains challenging in resource-limited settings, particularly in small or rural hospitals, where access to infectious disease (ID) specialists and clinical pharmacists is limited.^2^ The shortage of ID specialists in many healthcare systems represents a major barrier to the delivery of high-quality antimicrobial stewardship, leading to delayed specialist input and variability in antimicrobial prescribing practices.^3,4^

To address these challenges, telehealth-based approaches have increasingly been used to extend specialist expertise beyond traditional face-to-face consultation models.^5^ Telemedicine-based antimicrobial stewardship (tele-stewardship) is a promising strategy for supporting ASP implementation in hospitals without on-site ID specialists.^6^ Tele-stewardship is feasible and effective, with outcomes comparable to conventional in-person ASPs.^2,6^ For example, telehealth-based stewardship interventions have been associated with reductions in antimicrobial use and healthcare costs.^7,8^ However, these models often require dedicated personnel, secure remote access to electronic health records, and substantial local coordination, which may limit their scalability.

Among telehealth modalities, electronic consultation (eConsult) is popular as a practical, scalable model for delivering specialist expertise.^9^ eConsult refers to an asynchronous, provider-to-provider consultation conducted through a secure electronic platform, allowing clinicians to obtain specialist advice without requiring real-time interaction. Compared with synchronous telemedicine, eConsult offers greater flexibility and lower operational burden, thereby supporting resource-constrained settings. This consultation approach enables rapid turnaround of recommendations while ensuring structured documentation. Recent studies have suggested that ID-focused eConsult programs can improve clinical decision making and optimize antimicrobial use.^9^

The ExpertTWIN system, developed by the Shimadzu Corporation, is a web-based IDs consultation platform that can function as an ID eConsult system (Supplementary Material). When attending physicians encounter challenges related to ID diagnosis, antimicrobial selection, or infection control, they can consult registered ID specialists through this platform. Additionally, this system incorporates an input-assist function that helps users prepare consultation requests by supplementing essential patient information required for specialists to provide appropriate advice. The ExpertTWIN system complies with national guidelines for protecting personal information and the Act on the Protection of Personal Information, and implements appropriate measures to ensure security and management of patients’ personal information.

Despite growing global interest in ID eConsult models, evidence regarding their implementation and impact within the Japanese healthcare system remains limited. Specifically, the independent effect of eConsult-based interventions on antimicrobial stewardship outcomes in hospitals without on-site ID specialists remains unestablished.^10^ Therefore, this study aimed to evaluate the impact of an eConsult-based remote antimicrobial stewardship program using the ExpertTWIN system on antimicrobial consumption and resistance patterns, using a Bayesian structural time-series (BSTS) approach.

## Methods

### Study design and setting

This single-center retrospective observational study was conducted at Nagoya City University West Medical Center (NCUWMC), a 500-bed tertiary care hospital located in the Aichi Prefecture, Japan. Despite 33 clinical departments and primarily providing oncology care, it lacks an on-site ID consultation service and a dedicated ID department with board-certified ID specialists accredited by the Japanese Association for Infectious Diseases. The center admits approximately 13,000 inpatients annually. This study was conducted over a 24-month period from February 1, 2024 to January 31, 2026. Data were obtained from the ExpertTWIN system, Japan Surveillance for Infection Prevention and Healthcare Epidemiology (J-SIPHE) system,^11^ and electronic medical records of NCUWMC. Prior to implementation of the tele-stewardship program, two presentations were delivered to the antimicrobial stewardship team (AST) and relevant stakeholders at NCUWMC to facilitate institutional engagement and collaboration. Case consultations were provided by two board-certified ID specialists at Nagoya City University East Medical Center (NCUEMC) through the ExpertTWIN system.

### Interventions

#### Preintervention period (February 1, 2024 to January 31, 2025)

Once weekly, the AST at NCUWMC retrospectively reviewed cases in which designated antimicrobial agents had been used for ≥14 days, followed by feedback to the primary care team. Designated antimicrobial agents included carbapenems, piperacillin/tazobactam, ceftolozane/tazobactam, and anti-methicillin-resistant *Staphylococcus aureus* (MRSA) agents (vancomycin, teicoplanin, daptomycin, linezolid, and tedizolid). The AST consisted of two non-ID physicians, two pharmacists, and one nurse.

#### Postintervention period (February 1, 2025 to January 31, 2026)

In addition to the existing intervention, ExpertTWIN-based consultations were implemented. ExpertTWIN was used by off-site ID specialists to provide case-specific recommendations, whereas the on-site AST was responsible for reviewing these recommendations and communicating them to the primary care team. To ensure effective implementation of tele-stewardship through the ExpertTWIN platform, a standardized consultation template was shared in advance, outlining the required information to be included, such as medical history, vital signs and physical findings, allergy and past medical histories, prior antimicrobial administration, microbiological test results, presence of indwelling devices, and relevant laboratory data. Eligible cases were patients receiving carbapenem therapy for ≥7 days. These cases were identified by pharmacists at NCUWMC and referred for consultation as appropriate. The weekly target number of cases was initially set at 2–3 and was increased to 4–6 beginning on July 18, 2025. Furthermore, from July 18, 2025, patients who had received carbapenem therapy for 3–6 days were also considered eligible for consultation when the weekly target number of cases was not met. Consultation cases were reviewed by ID specialists at NCUEMC, and responses were provided within 24 hours through the ExpertTWIN system. For each case, recommendations from ID specialists were reviewed at AST conferences at NCUWMC, and pharmacists subsequently conveyed specific suggestions to the primary care team (see Supplementary Material). When additional clarification or educational opportunities were identified, ID specialists provided supplementary explanations to support clinical decision making.

### Outcome measures

The primary outcome was the change in days of therapy (DOTs) for intravenous carbapenems (imipenem/cilastatin, meropenem, and doripenem), expressed as DOTs per 100 patient-days per month. Carbapenems were selected as the primary outcome owing to their broad spectrum and the critical importance of ensuring their appropriate use.

Secondary outcomes included DOTs for four antipseudomonal agents (piperacillin/tazobactam, cefepime, cefozopran, and ceftolozane/tazobactam), DOTs for six narrow-spectrum agents (penicillin G, ampicillin, ampicillin/sulbactam, cefazolin, cefmetazole, and flomoxef), and DOTs for five anti-MRSA agents (vancomycin, teicoplanin, daptomycin, linezolid, and tedizolid). Additionally, total DOTs and antimicrobial costs for all intravenous and oral agents were evaluated. Additional outcomes included the incidence of hospital-acquired resistant organisms, incidence of *Clostridioides difficile* infection (CDI), number of culture specimens obtained per 1,000 patient-days, all-cause in-hospital mortality within 30 days, length of hospital stay, appropriateness of antimicrobial therapy assessed using the ABCDEFHIT criteria, and the rate and acceptance of AST recommendations (Supplementary Material).

### Statistical analysis

Between February 1, 2024, and January 31, 2026, BSTS analyses were conducted for the primary and secondary outcomes.^12^ Given the availability of 12 preintervention time points for model training, the common BSTS model was specified as a univariate time-series model with a local-level component. Sensitivity analyses for the primary outcome were performed using three alternative specifications: (1) a local-level model with a 12-month seasonal component, (2) a local linear trend model, and (3) a Poisson BSTS model with a local-level component (offset by patient-days). No external covariates were included; therefore, counterfactual predictions relied solely on the preintervention time-series history of each outcome.

Model parameters were estimated using Markov chain Monte Carlo (MCMC) sampling with 8,000 iterations and no burn-in period. All other settings were based on the default specification of the *CausalImpact* package (see Brodersen et al.^12^ for details of the prior distributions). MCMC convergence was assessed using the potential scale reduction factor (R-hat) and effective sample size, calculated from four chains initiated with different random seeds. Convergence was considered adequate when R-hat was <1.01 and ESS was >400. The average intervention effect was summarized using the posterior mean, a 95% credible interval (CrI), and the posterior probability (PP) of the hypothesized effect direction.

For antimicrobial purchase costs, a *t*-test was used to compare values before and after the intervention. Statistical significance was set at *P* < .05. All analyses were performed using R software (version 4.2.0; R Foundation for Statistical Computing, Vienna, Austria). No data were missing.

### Ethical considerations

The study protocol was approved by the Institutional Review Board of Nagoya City University (approval number: 60-24-0138), and conformed to the Declaration of Helsinki (2024). The requirement for informed consent was waived because this study only used clinical practice data.

## Results

In total, 27,863 patients were hospitalized: 13,501 during the preintervention period and 14,362 during the postintervention period, corresponding to 123,969 and 130,341 patient-days, respectively. There were 21 cases of antimicrobial supply restrictions during the study period (Supplementary Table 1). Two carbapenems were subject to continuous usage restrictions throughout the study period; however, no order suspensions occurred. During the preintervention and postintervention periods, the existing offline ASP conducted by the AST at NCUWMC provided 184 and 150 feedback reports, respectively, for targeted antimicrobial use. During the intervention period, the tele-stewardship program conducted 145 case reviews of antimicrobial use. Case reviews were most frequently conducted in the Departments of Gastroenterology (45 cases, 31.0%) and Hematology/Oncology (38 cases, 26.2%). Among patients who underwent case review, 29 (20.0%) had hematologic malignancies, 46 (31.7%) had solid tumors, four (2.8%) had both, and 66 (45.5%) had non-malignant conditions. Based on assessments by ID specialists at NCUEMC, two cases (1.4%) were judged to represent appropriate use of the targeted antimicrobial agents (category A, 0 cases; B, two cases), whereas 143 cases (98.6%) were determined to represent inappropriate use (category C, four cases; D, 69 cases; E, three cases; F, 0 cases; H, 67 cases; I, 0 cases; T, 0 cases) (Supplementary Table 2). The median time required to prepare responses was 6 minutes 29 seconds (range, 52 s to 27 min 5 s) and the median response time was 44 minutes 26 seconds (range, 3 min 35 s to 21 h 4 min 21 s). Among the cases reviewed through the tele-stewardship program, 214 recommendations regarding antimicrobial prescribing or diagnostic testing were provided, of which 135 (63.1%) were accepted.

### Antibiotic consumption

**Figure 1.**
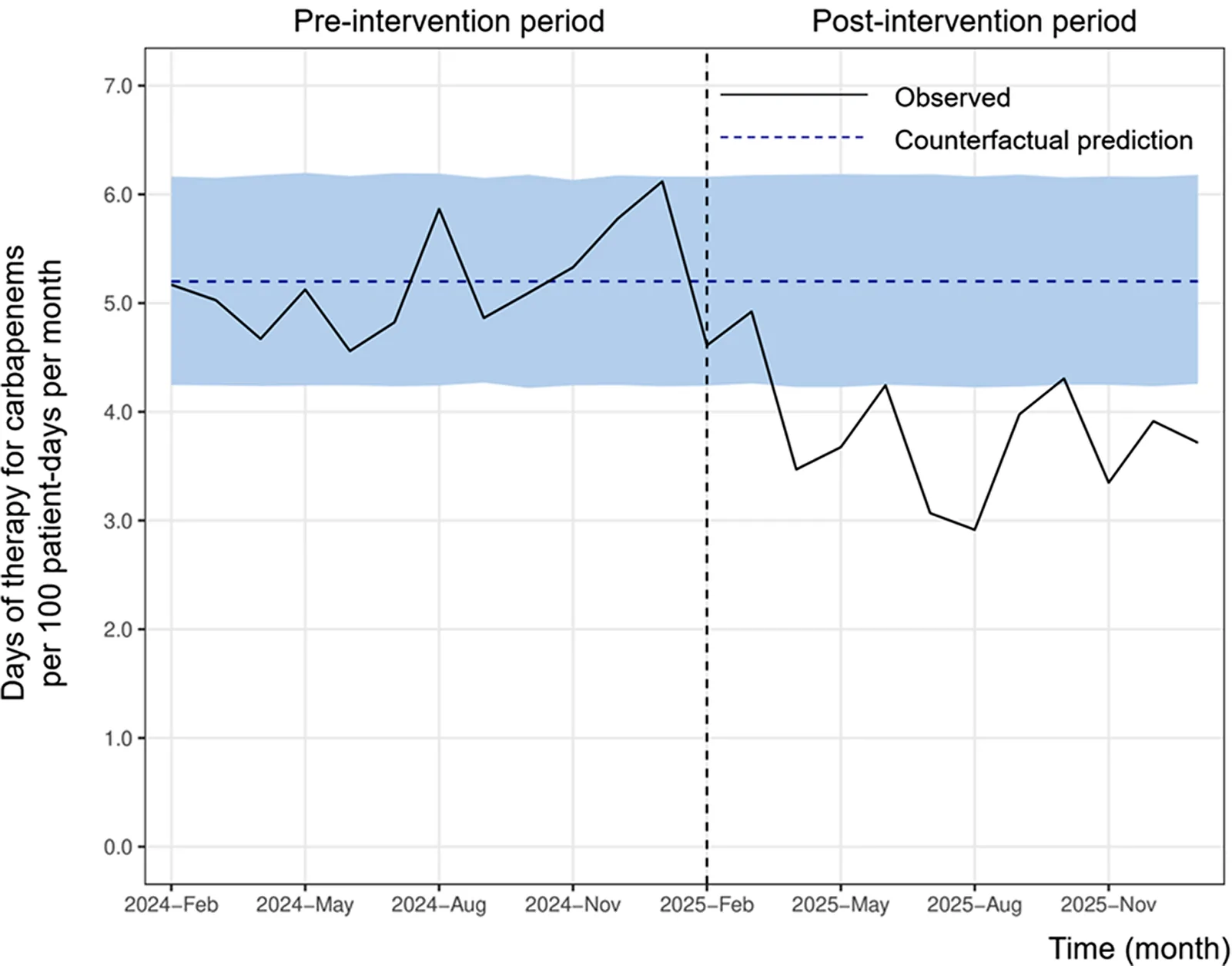
Days of therapy for carbapenems per 100 patient-days per month. Days of therapy (DOTs) for three carbapenems (imipenem/cilastatin, meropenem, and doripenem) per 100 patient-days per month are shown. The study comprised two phases: the preintervention period (February 1, 2024 to January 31, 2025) and postintervention period (February 1, 2025 to January 31, 2026). The light blue shaded area represents the 95% credible interval (CrI) around the predicted values. Carbapenem use decreased by 1.4 DOTs per 100 patient-days per month (average effect, −1.4 [95% CrI, −1.7 to −1.0]; posterior probability [PP] for reduction, 99.9%).

**Figure 2.**
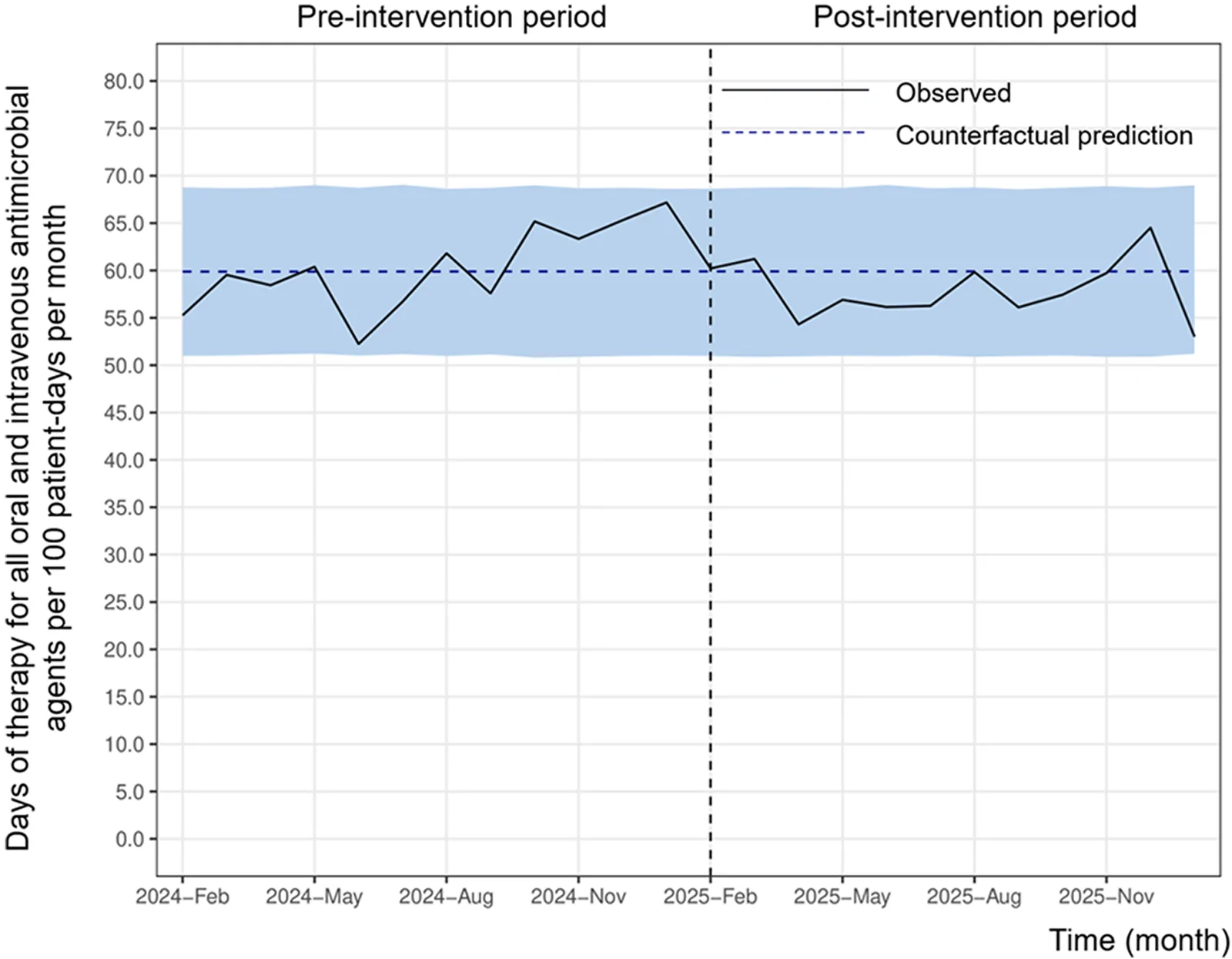
Days of therapy for all oral and intravenous antimicrobial agents per 100 patient-days per month. Days of therapy (DOTs) for all oral and intravenous antimicrobial agents per 100 patient-days per month are shown. The study comprised two phases: the preintervention period (February 1, 2024 to January 31, 2025) and postintervention period (February 1, 2025 to January 31, 2026). The light blue shaded area represents the 95% credible interval (CrI) around the predicted values. Total DOTs for all oral and intravenous antimicrobial agents showed evidence of a reduction (average effect, −1.9 [95% CrI, −5.3 to 1.6]; PP for reduction, 86.7%).

Table 1 summarizes the changes in DOTs for each antibiotic between the preintervention and postintervention periods. Carbapenem DOTs decreased (Figure 1; −1.4 [95% CrI, −1.7 to −1.0]; PP for reduction, 99.9%). DOTs showed evidence of increases for four antipseudomonal agents (0.3 [95% CrI, −0.1 to 0.7]; PP for increase, 93.3%) and six narrow-spectrum agents (0.6 [95% CrI, −0.1 to 1.4]; PP for increase, 94.8%) and of a reduction for anti-MRSA agents (−0.2 [95% CrI, −0.6 to 0.1]; PP for reduction, 92.1%). Total oral and intravenous antimicrobial DOTs showed evidence of a reduction (Figure 2; −1.9 [95% CrI, −5.3 to 1.6]; PP for reduction, 86.7%). All models showed satisfactory MCMC convergence (Supplementary Table 4). Sensitivity analyses yielded effects in the same direction, although the estimated magnitude and precision varied across model specifications (Supplementary Table 3).

**Table 1.** Bayesian structural time-series analysis of antimicrobial use, incidence of antimicrobial-resistant microorganisms, laboratory metrics, and patient outcomes Table 1 long description.

	Preintervention mean	Postintervention mean	Average effect	95% CrI	PP of the hypothesized effect direction (%)
**Use of antimicrobial agents**					
DOTs for carbapenems^a^ per 100 patient-days per month	5.2 (0.5)	3.8 (0.6)	−1.4	(−1.7, −1.0)	99.9
DOTs for antipseudomonal agents^b^ per 100 patient-days per month	4.9 (0.5)	5.2 (0.7)	0.3	(−0.1, 0.7)	93.3
DOTs for narrow-spectrum antibiotics^c^ per 100 patient-days per month	14.3 (1.0)	14.9 (0.7)	0.6	(−0.1, 1.4)	94.8
DOTs for anti-MRSA antimicrobial agents^d^ per 100 patient-days per month	2.8 (0.4)	2.5 (0.5)	−0.2	(−0.6, 0.1)	92.1
DOTs for all intravenous antimicrobial agents per 100 patient-days per month	36.8 (2.1)	36.0 (1.9)	−0.7	(−2.3, 0.9)	81.9
DOTs for all oral antimicrobial agents per 100 patient-days per month	23.4 (3.2)	22.0 (2.7)	−1.2	(−3.6, 1.3)	84.0
DOTs for total intravenous and oral antimicrobial agents per 100 patient-days per month	60.2 (4.5)	58.0 (3.2)	−1.9	(−5.3, 1.6)	86.7
**Incidence of antimicrobial-resistant microorganisms**					
CRE per 1,000 patient-days per month^1^	0 (0)	0.01 (0.03)	–	–	–
Third-generation cephalosporin-resistant *Escherichia coli* per 1,000 patient-days per month	0.42 (0.17)	0.63 (0.24)	0.20	(0.06, 0.33)	99.7
Third-generation cephalosporin-resistant *Klebsiella pneumoniae* per 1,000 patient-days per month^1^	0 (0)	0.01 (0.03)	–	–	–
CDI per 1,000 patient-days per month	0.15 (0.12)	0.12 (0.10)	−0.01	(−0.10, 0.08)	61.7
CRPA per 1,000 patient-days per month	0.11 (0.07)	0.08 (0.08)	−0.04	(−0.09, 0.01)	93.7
Two-drug-resistant *Pseudomonas aeruginosa* per 1,000 patient-days per month	0.04 (0.07)	0 (0)	−0.04	(−0.10, 0.02)	90.3
MDRP per 1,000 patient-days per month^1^	0 (0)	0 (0)	–	–	–
MRSA per 1,000 patient-days per month	1.3 (0.3)	1.1 (0.3)	−0.14	(−0.40, 0.12)	86.8
**Laboratory metric**					
Culture submission rate per 1,000 patient-days per month	82.4 (8.0)	86.7 (4.3)	3.9	(−2.4, 9.8)	89.3
**Patient outcome**					
All-cause in-hospital mortality (%)	2.2 (0.5)	2.1 (0.4)	−0.09	(−0.47, 0.29)	67.7
Average length of hospital stay (days)	9.2 (0.5)	9.1 (0.7)	−0.12	(−0.52, 0.26)	73.3

Values are presented as mean (standard deviation).

CrI, credible interval; CDI, *Clostridioides difficile* infection; CRE, carbapenem-resistant Enterobacterales; CRPA, carbapenem-resistant *Pseudomonas aeruginosa*; DOTs, days of therapy; MDRP, multidrug-resistant *Pseudomonas aeruginosa*; MRSA, methicillin-resistant *Staphylococcus aureus*; PP, Posterior probability.

a Imipenem/cilastatin, meropenem, and doripenem.

b Cefepime, cefozopran, piperacillin/tazobactam, and ceftolozane/tazobactam.

c Penicillin G, ampicillin, ampicillin/sulbactam, cefazolin, cefmetazole, and flomoxef.

d Vancomycin, teicoplanin, daptomycin, linezolid, and tedizolid.

1 Because the preintervention data were zero, the causal impact analysis could not be applied.

### Incidences of antimicrobial-resistant microorganisms and CDI

The incidences of carbapenem-resistant *P. aeruginosa* (−0.04 [95% CrI, −0.09 to 0.01]; PP for reduction, 93.7%), two-drug–resistant *P. aeruginosa* (−0.04 [95% CrI, −0.10 to 0.02]; PP for reduction, 90.3%), and MRSA (−0.14 [95% CrI, −0.40 to 0.12]; PP for reduction, 86.8%) showed evidence of reductions, whereas that of third-generation cephalosporin-resistant *Escherichia coli* increased (0.20 [95% CrI, 0.06–0.33]; PP for increase, 99.7%; Table 1). There was no clear evidence of a reduction in CDI (−0.01 [95% CrI, −0.10 to 0.08]; PP for reduction, 61.7%).

### Cost of antimicrobial agents

The actual and adjusted costs of carbapenems and all oral antimicrobial agents per patient day decreased significantly (Table 2; carbapenems actual cost, *P* < .001; carbapenems adjusted cost, *P* < .001; oral antimicrobial agents actual cost, *P* = .04; oral antimicrobial agents adjusted cost, *P* = .02). However, no significant changes were observed in total antimicrobial costs.

**Table 2.** Antimicrobial purchase costs per patient-day during the pre and postintervention periods Table 2 long description.

	Preintervention period (February 2024 to January 2025)	Postintervention period (February 2025 to January 2026)	*P*-value
**Actual cost**			
CARs per patient-day, USD	0.83 (0.08)	0.61 (0.08)	<.001
All intravenous antimicrobial agents per patient-day, USD	4.27 (0.31)	4.31 (0.33)	.79
All oral antimicrobial agents per patient-day, USD	0.14 (0.07)	0.09 (0.02)	.04
All intravenous and oral antimicrobial agents per patient-day, USD	4.41 (0.34)	4.40 (0.33)	.93
**Adjusted cost^a^**			
CARs per patient-day, USD	0.75 (0.07)	0.54 (0.07)	<.001
All intravenous antimicrobial agents per patient-day, USD	5.00 (0.41)	4.96 (0.43)	.82
All oral antimicrobial agents per patient-day, USD	0.18 (0.07)	0.13 (0.02)	.02
All intravenous and oral antimicrobial agents per patient-day, USD	5.18 (0.44)	5.09 (0.43)	.60

Values are presented as mean (standard deviation).

CAR, carbapenem; USD, United States dollar.

The cost calculation was based on an exchange rate of 150 yen per USD, as of March 2025.

a The adjusted purchase costs were calculated on the basis of the actual purchase costs (considering the cost of switching from branded to generic products and changes in drug prices) and prices of branded agents.

### Culture specimen submissions

The culture specimen submission rate showed evidence of an increase (3.9 [95% CrI, −2.4–9.8]; PP for increase, 89.3%; Table 1).

### All-cause in-hospital mortality and length of hospital stay

There was no clear evidence of a reduction in either all-cause in-hospital mortality (−0.09 [95% CrI, −0.47 to 0.29]; PP for reduction, 67.7%) or average length of hospital stay (−0.12 [95% CrI, −0.52 to 0.26]; PP for reduction, 73.3%) after the intervention (Table 1).

## Discussion

We conducted one of the first evaluations of a tele-stewardship program using a web-based consultation platform developed in Japan. This targeted strategy was embedded within an existing AST framework and focused on patients receiving prolonged carbapenem therapy, thereby enriching the reviewed cohort with cases of potentially inappropriate use. Following implementation, carbapenem use decreased; anti-MRSA agent use, total antimicrobial use, and the incidences of several antimicrobial-resistant organisms showed evidence of reductions; and culture specimen submission showed evidence of an increase. However, third-generation cephalosporin-resistant *E. coli* increased, and there was no clear evidence of changes in mortality or length of stay. These findings suggest that targeted interventions may influence practice beyond directly reviewed cases.

The program used an integrated tele-stewardship model that complemented the existing offline ASP through close coordination with the on-site AST.^2^ Unlike fully remote models, in which off-site specialists directly review electronic records, or collaborative models led mainly by on-site staff with remote support, recommendations were relayed through the on-site AST.^2^ This approach may support acceptance, sustainability, and staff education.^8,10,13^ Although NCUWMC lacked on-site ID specialists and ASP-dedicated pharmacists, its existing AST enabled pharmacists to assume expanded roles.

We implemented the tele-stewardship program using ExpertTWIN, a fee-based platform for asynchronous specialist consultation. Unlike models requiring remote electronic health record access or videoconferencing, eConsult allows flexible responses without real-time coordination, reducing practical barriers related to scheduled meetings.^5,8,14,15^ The short response preparation and response times observed in this study supported operational feasibility. Similar web-based tele-stewardship platforms have been reported in Brazil.^16,17^ ExpertTWIN also offers a structured chat log that enhances recommendation traceability and an input-support system that facilitates consultation. Future development of ExpertTWIN could support agentic AI–enabled eConsult services, subject to prospective validation and continued oversight by ID specialists. Overall, ExpertTWIN has potential for broader implementation in Japanese hospitals lacking ID specialists. Before implementation, however, each medical center should assess how ExpertTWIN would integrate with its existing stewardship program and evaluate its anticipated net financial impact.

Carbapenem use decreased after implementation, while anti-MRSA agent use and total oral and intravenous antimicrobial use showed evidence of reductions; antipseudomonal-agent and narrow-spectrum antibiotic use showed evidence of increases. The concurrent decrease in carbapenem use and increase in antipseudomonal-agent use may partly reflect carbapenem-sparing substitution or de-escalation following stewardship recommendations, although the aggregate data did not allow us to distinguish this explanation from changes in clinical indications for antipseudomonal therapy. Anti-MRSA agents were not direct intervention targets, but recommendations often addressed the overall antimicrobial regimen. No major ASP policy changes occurred during the postintervention period. Two carbapenems were restricted throughout both periods without order suspension, making differential restrictions an unlikely sole explanation, although residual supply effects cannot be excluded. There was no clear evidence of changes in mortality or length of stay; these findings do not establish safety or noninferiority.

The incidences of carbapenem-resistant *Pseudomonas aeruginosa*, two-drug-resistant *P. aeruginosa*, and MRSA showed evidence of reductions, consistent with previous studies of on-site ASPs.^18–21^ By contrast, third-generation cephalosporin-resistant *Escherichia coli* increased. This increase may reflect the rising community prevalence of extended-spectrum *β*-lactamase–producing *E. coli* in Japan.^22^ Enhanced detection may also have contributed, as culture specimen submission showed evidence of an increase. Although this finding is consistent with improved diagnostic practices, causality cannot be established. These findings suggest that eConsult-based tele-stewardship may influence both prescribing and diagnostic stewardship.

Most reviewed cases were deemed inappropriate, mainly requiring discontinuation or de-escalation. This high proportion likely reflects pharmacist-led, duration-based screening of carbapenem therapy, which enriched the cohort with cases requiring modification, rather than the hospitalwide prevalence of inappropriate prescribing. Comparable preintervention data were unavailable because appropriateness was not systematically assessed using the ABCDEFHIT criteria. The predominance of discontinuation and de-escalation recommendations also suggests that reviews occurred relatively late. Earlier review, around days 3–4 when culture results are often available, may improve the program. By comparison, previous studies of daily on-site ASPs reported that approximately 45%–50% of prescriptions were appropriate.^20,21^ A predefined day 3–4 trigger may be feasible within the asynchronous ExpertTWIN workflow, with its input-assist function supporting structured submission and rapid review; however, prospective evaluation is needed. In this study, 63.1% of the recommendations were accepted. As tele-stewardship becomes more fully integrated into routine practice, the acceptance rate may increase further.

Actual and adjusted costs of carbapenems and oral antimicrobial agents were substantially reduced. Although oral agents were not directly targeted, frequent discontinuation recommendations suggest that intravenous-to-oral switching was not actively promoted, possibly explaining the reduced oral use; no related policy changes occurred. Total antimicrobial costs changed little but showed a decreasing trend.

This study has limitations. First, the single-center design and the high proportion of patients with malignancies among the reviewed cases may limit generalizability.^23^ Second, consultations were provided by few ID specialists; because ExpertTWIN is intended for broader national use, multicenter validation is needed. Third, antimicrobial supply restrictions occurred. Although two carbapenems were restricted throughout both periods without order suspensions, residual effects and substitution with other antipseudomonal agents cannot be excluded. Fourth, preimplementation presentations were limited to program personnel rather than hospitalwide prescribers, likely limiting a direct Hawthorne effect; however, increased awareness among program personnel may have independently influenced stewardship activities. Fifth, the intervention was brief and targeted limited agents, and broader, longer implementation may yield different effects. Finally, the limited number of preintervention time points warrants cautious interpretation of the BSTS results.

Collectively, this study found that an integrated, web-based ID eConsult tele-stewardship program was associated with decreased carbapenem use, evidence of reductions in several antimicrobial-use and resistant-organism outcomes, and evidence of increased culture specimen submission in a hospital without on-site ID specialists. However, third-generation cephalosporin-resistant *Escherichia coli* increased, and there was no clear evidence of changes in mortality or length of stay. This model may support antimicrobial prescribing and diagnostic practices in similar settings. Multicenter studies with longer follow-up are needed to assess its generalizability and longer-term effects on antimicrobial resistance.

## Supporting information

10.1017/ash.2026.10851.sm001 Itoh et al. supplementary material 1 Itoh et al. supplementary material

10.1017/ash.2026.10851.sm002 Itoh et al. supplementary material 2 Itoh et al. supplementary material

10.1017/ash.2026.10851.sm003 Itoh et al. supplementary material 3 Itoh et al. supplementary material

## Supplementary material

The supplementary material for this article can be found at https://doi.org/10.1017/ash.2026.10851.

## Research transparency and reproducibility

The data sets used and analyzed in the current study are available from the corresponding author upon reasonable request.

## Table 1. Long description

A table with 12 rows and 5 columns comparing antimicrobial use, incidence of antimicrobial-resistant microorganisms, laboratory metrics, and patient outcomes preintervention and postintervention. The columns are Preintervention mean, Postintervention mean, Average effect, 95% CrI, and PP of the hypothesized effect direction (percent). The table includes data on DOTs for various antimicrobial agents, incidence of resistant microorganisms, laboratory metrics, and patient outcomes. Notable trends include a decrease in carbapenem DOTs and an increase in DOTs for antipseudomonal and narrow-spectrum agents. The incidence of third-generation cephalosporin-resistant Escherichia coli increased, while other resistant microorganisms showed varied changes. Laboratory metrics and patient outcomes also showed some changes postintervention.

Navigate back to Table 1..

## Table 2. Long description

A table comparing antimicrobial purchase costs per patient-day during pre and postintervention periods. The table has 2 main sections: Actual cost and Adjusted cost, each with 4 rows and 3 columns. The columns are labeled Preintervention period (February 2024 to January 2025), Postintervention period (February 2025 to January 2026), and P-value. The rows under Actual cost are CARs per patient-day in USD, All intravenous antimicrobial agents per patient-day in USD, All oral antimicrobial agents per patient-day in USD, and All intravenous and oral antimicrobial agents per patient-day in USD. The rows under Adjusted cost are the same as under Actual cost. Each row contains values for the pre and postintervention periods and a P-value indicating the significance of the change. Notable trends include a significant decrease in the actual and adjusted costs of CARs and all oral antimicrobial agents per patient-day, while no significant changes were observed in the total antimicrobial costs.

Navigate back to Table 2..
