# Supplementary material for: Impact of an eConsult-based antimicrobial stewardship program using the ExpertTWIN system: a Bayesian structural time-series analysis
Source: Antimicrob Steward Healthc Epidemiol. 2026 Sep 7;6(1):e255. doi: 10.1017/ash.2026.10851 (PMC13760392; doi:10.1017/ash.2026.10851)
Supplement: Supplementary file 1 [file S2732494X26108511sup001.docx]

**Supplementary Figures**

**Supplementary Figure 1.** Days of therapy for antipseudomonal agents per 100 patient-days per month.

Days of therapy (DOTs) for four antipseudomonal agents (piperacillin/tazobactam, cefepime, cefozopran, and ceftolozane/tazobactam) per 100 patient-days per month are shown. The study comprised two phases: the pre-intervention period (February 1, 2024 to January 31, 2025) and the post-intervention period (February 1, 2025 to January 31, 2026). The light blue shaded area represents the 95% credible interval (CrI) around the predicted values. DOTs for antipseudomonal agents showed evidence of an increase (average effect, 0.3 [95% CrI, −0.1 to 0.7]; posterior probability [PP] for increase, 93.3%).

**Supplementary Figure 2.** Days of therapy for narrow-spectrum antibiotics per 100 patient-days per month.

Days of therapy (DOTs) for six narrow-spectrum antibiotics (penicillin G, ampicillin, ampicillin/sulbactam, cefazolin, cefmetazole, and flomoxef) per 100 patient-days per month are shown. The study comprised two phases: the pre-intervention period (February 1, 2024 to January 31, 2025) and the post-intervention period (February 1, 2025 to January 31, 2026). The light blue shaded area represents the 95% credible interval (CrI) around the predicted values. DOTs for narrow-spectrum antibiotics showed evidence of an increase (average effect, 0.6 [95% CrI, −0.1 to 1.4]; PP for increase, 94.8%).

**Supplementary Figure 3.** Days of therapy for anti-methicillin-resistant *Staphylococcus aureus* agents per 100 patient-days per month.

Days of therapy (DOTs) for five anti-methicillin-resistant *Staphylococcus aureus* (MRSA) antimicrobial agents (vancomycin, teicoplanin, daptomycin, linezolid, and tedizolid) per 100 patient-days per month are shown. The study comprised two phases: the pre-intervention period (February 1, 2024 to January 31, 2025) and the post-intervention period (February 1, 2025 to January 31, 2026). The light blue shaded area represents the 95% credible interval (CrI) around the predicted values. DOTs for anti-MRSA agents showed evidence of a reduction (average effect, −0.2 [95% CrI, −0.6 to 0.1]; PP for reduction, 92.1%).

**Supplementary Figure 4.** Days of therapy for all intravenous antimicrobial agents per 100 patient-days per month.

Days of therapy (DOTs) for all intravenous antimicrobial agents per 100 patient-days per month are shown. The study comprised two phases: the pre-intervention period (February 1, 2024 to January 31, 2025) and the post-intervention period (February 1, 2025 to January 31, 2026). The light blue shaded area represents the 95% credible interval (CrI) around the predicted values. Total intravenous antimicrobial DOTs showed evidence of a reduction (average effect, −0.7 [95% CrI, −2.3 to 0.9]; PP for reduction, 81.9%).

**Supplementary Figure 5.** Days of therapy for all oral antimicrobial agents per 100 patient-days per month.

Days of therapy (DOTs) for all oral antimicrobial agents per 100 patient-days per month are shown. The study comprised two phases: the pre-intervention period (February 1, 2024 to January 31, 2025) and the post-intervention period (February 1, 2025 to January 31, 2026). The light blue shaded area represents the 95% credible interval (CrI) around the predicted values. Total oral antimicrobial DOTs showed evidence of a reduction (average effect, −1.2 [95% CrI, −3.6 to 1.3]; PP for reduction, 84.0%).

**Supplementary Figure 6.** Incidence of the third-generation cephalosporin-resistant *Escherichia coli* per 1,000 patient-days per month.

The monthly incidence of the third-generation cephalosporin-resistant *Escherichia coli* per 1,000 patient-days is shown. The study comprised two phases: the pre-intervention period (February 1, 2024 to January 31, 2025) and the post-intervention period (February 1, 2025 to January 31, 2026). The light blue shaded area represents the 95% credible interval (CrI) around the predicted values. The incidence increased (average effect, 0.20 [95% CrI, 0.06 to 0.33]; PP for increase, 99.7%).

**Supplementary Figure 7.** Incidence of *Clostridioides difficile* infection per 1,000 patient-days per month.

The monthly incidence of *Clostridioides difficile* infection (CDI) per 1,000 patient-days is shown. The study comprised two phases: the pre-intervention period (February 1, 2024 to January 31, 2025) and the post-intervention period (February 1, 2025 to January 31, 2026). The light blue shaded area represents the 95% credible interval (CrI) around the predicted values. There was no clear evidence of a reduction in CDI incidence (average effect, −0.01 [95% CrI, −0.10 to 0.08]; PP for reduction, 61.7%).

**Supplementary Figure 8.** Incidence of the carbapenem-resistant *Pseudomonas aeruginosa* per 1,000 patient-days per month.

The monthly incidence of the carbapenem-resistant *Pseudomonas aeruginosa* (CRPA) per 1,000 patient-days is shown. The study comprised two phases: the pre-intervention period (February 1, 2024 to January 31, 2025) and the post-intervention period (February 1, 2025 to January 31, 2026). The light blue shaded area represents the 95% credible interval (CrI) around the predicted values. CRPA incidence showed evidence of a reduction (average effect, −0.04 [95% CrI, −0.09 to 0.01]; PP for reduction, 93.7%).

**Supplementary Figure 9.** Incidence of the two-drug-resistant *Pseudomonas aeruginosa* per 1,000 patient-days per month.

The monthly incidence of the two-drug-resistant *Pseudomonas aeruginosa* per 1,000 patient-days is shown. The study comprised two phases: the pre-intervention period (February 1, 2024 to January 31, 2025) and the post-intervention period (February 1, 2025 to January 31, 2026). The light blue shaded area represents the 95% credible interval (CrI) around the predicted values. The incidence showed evidence of a reduction (average effect, −0.04 [95% CrI, −0.10 to 0.02]; PP for reduction, 90.3%).

**Supplementary Figure 10.** Incidence of the methicillin-resistant *Staphylococcus aureus* per 1,000 patient-days per month.

The monthly incidence of the methicillin-resistant *Staphylococcus aureus* (MRSA) per 1,000 patient-days is shown. The study comprised two phases: the pre-intervention period (February 1, 2024 to January 31, 2025) and the post-intervention period (February 1, 2025 to January 31, 2026). The light blue shaded area represents the 95% credible interval (CrI) around the predicted values. MRSA incidence showed evidence of a reduction (average effect, −0.14 [95% CrI, −0.40 to 0.12]; PP for reduction, 86.8%).

**Supplementary Figure 11.** Monthly culture specimen submission rate per 1,000 patient-days.

The monthly culture specimen submission rate per 1,000 patient-days is shown. The study comprised two phases: the pre-intervention period (February 1, 2024 to January 31, 2025) and the post-intervention period (February 1, 2025 to January 31, 2026). The light blue shaded area represents the 95% credible interval (CrI) around the predicted values. The culture specimen submission rate showed evidence of an increase (average effect, 3.9 [95% CrI, −2.4 to 9.8]; PP for increase, 89.3%).

**Supplementary Figure 12.** All-cause in-hospital mortality.

The monthly all-cause in-hospital mortality rate is shown. The study comprised two phases: the pre-intervention period (February 1, 2024 to January 31, 2025) and the post-intervention period (February 1, 2025 to January 31, 2026). The light blue shaded area represents the 95% credible interval (CrI) around the predicted values. There was no clear evidence of a reduction in all-cause in-hospital mortality (average effect, −0.09 [95% CrI, −0.47 to 0.29]; PP for reduction, 67.7%).

**Supplementary Figure 13.** Average length of hospital stay.

The monthly average length of hospital stay is shown. The study comprised two phases: the pre-intervention period (February 1, 2024 to January 31, 2025) and the post-intervention period (February 1, 2025 to January 31, 2026). The light blue shaded area represents the 95% credible interval (CrI) around the predicted values. There was no clear evidence of a reduction in the average length of hospital stay (average effect, −0.12 [95% CrI, −0.52 to 0.26]; PP for reduction, 73.3%).
