# Supplementary material for: Impact of an eConsult-based antimicrobial stewardship program using the ExpertTWIN system: a Bayesian structural time-series analysis
Source: Antimicrob Steward Healthc Epidemiol. 2026 Sep 7;6(1):e255. doi: 10.1017/ash.2026.10851 (PMC13760392; doi:10.1017/ash.2026.10851)
Supplement: Supplementary file 2 [file S2732494X26108511sup002.docx]

**Supplementary Material**

**Title: Impact of an eConsult-based antimicrobial stewardship program using the ExpertTWIN system: a Bayesian structural time-series analysis**

**Infectious diseases management support system (ExpertTWIN)**

ExpertTWIN is a secure, web-based consultation platform designed to assist remote ID management. The system is not subject to Japan’s Pharmaceuticals and Medical Devices Act. AST members at the participating hospital were able to submit consultation requests when they encountered diagnostic or therapeutic challenges related to IDs. Consultations were entered through a chat-based interface, allowing detailed clinical information to be shared efficiently. Patient information, including laboratory results and clinical summaries, could be transmitted securely in a structured format. When additional visual information was required, relevant images (e.g., wound photographs and imaging results) could be uploaded. Once a consultation request was submitted, the assigned ID specialist received an automated email notification. The requesting physician was notified by email once the specialist’s response became available. All communications were securely stored within the system and could be reviewed by both parties to ensure continuity of care and facilitate subsequent evaluation. To support data entry, the system also includes an auxiliary question-guidance function. When patient information is entered, a medical language processing engine and dictionary-based recognition system automatically identify relevant clinical elements and generate additional questions that specialists commonly require for consultation. The relative contribution of individual ExpertTWIN features to the eConsult workflow was not evaluated in this study. No fine-tuning of the medical language processing engine or dictionary-based recognition system was performed during the post-intervention period.

**Supplementary Methods**

Assessment and acceptance rate of antimicrobial stewardship team recommendations

In the telemedicine-based antimicrobial stewardship (tele-stewardship) program, the appropriateness of antimicrobial use was assessed, and the acceptance rate of recommendations was calculated. Infectious disease specialists at Nagoya City University East Medical Center qualitatively assessed the appropriateness of specific antimicrobial use for each case at the time of consultation using the ABCDEFHIT criteria,^1, 2^ based on the culture results, treatment course, and duration. The ABCDEFHIT criteria are a qualitative framework for evaluating antimicrobial use. It categorizes prescriptions as appropriate (A, B) or inappropriate (C-T). Category A indicates fully appropriate selection and dosing, and B denotes acceptable therapy with room for minor improvement. C reflects inadequate or missing culture submission, D suggests that broad-spectrum agents should be de-escalated, and E indicates insufficient antimicrobial coverage requiring escalation. F reflects inappropriate dosing or administration, H recommends discontinuation when therapy goals are achieved or risks arise, I identifies missing documentation of indication, and T serves as a time-out prompt to review culture results or reassess the need for continued therapy.

For patients in whom the tele-stewardship program determined that modification of antimicrobial therapy or additional testing was warranted, feedback was provided by pharmacists at Nagoya City University West Medical Center to the primary care team via telephone or electronic medical records. All patients subject to audit were monitored until completion of the designated antimicrobial therapy or for the period deemed necessary by the antimicrobial stewardship team (AST). In cases where discrepancies arose between the recommendations of the tele-stewardship program and the judgment of the primary care team, adjustments were made through discussion, with the final decision left to the discretion of the primary care team. Acceptance was defined as implementation of recommendations to modify or discontinue antimicrobial therapy within the timeframe specified by the AST, or completion of recommended additional diagnostic testing. De-escalation or discontinuation was counted as an accepted intervention only when the recommendation was implemented. Diagnostic tests that had already been ordered at the time of recommendation were not included in the count. When multiple tests were proposed simultaneously, they were recorded as a single proposal, and if one or more were accepted, the case was counted as one accepted intervention.

Days of therapy for antipseudomonal agents

The days of therapy (DOTs) per 100 patient-days per month were calculated for four intravenous antipseudomonal agents (piperacillin/tazobactam, cefepime, cefozopran, and ceftolozane/tazobactam). These agents were selected as outcomes because, similar to carbapenems, they have a broad antimicrobial spectrum.

Days of therapy for narrow-spectrum antimicrobial agents

Penicillin G, ampicillin, ampicillin/sulbactam, cefazolin, cefmetazole, and flomoxef were classified as narrow-spectrum intravenous antimicrobial agents, and the DOTs per 100 patient-days per month were calculated for these six agents. These agents were selected as indicators for de-escalation from broad-spectrum antimicrobial agents.

Days of therapy for anti-methicillin-resistant *Staphylococcus aureus* antimicrobial agents

Vancomycin, teicoplanin, daptomycin, linezolid, and tedizolid were classified as intravenous anti-methicillin-resistant *Staphylococcus aureus* **(**MRSA) agents, and the DOTs per 100 patient-days per month were calculated for these five agents. The DOTs per 100 patient-days per month were calculated for all intravenous antimicrobial agents, all oral antimicrobial agents, and the combined total of intravenous and oral antimicrobial agents. These indicators were used to evaluate the overall impact of the intervention on inpatient antimicrobial use.

Incidences of hospital-acquired resistant microorganisms and *Clostridioides difficile* infection

As outcome indicators of the antimicrobial stewardship program, the incidences of hospital-acquired resistant microorganisms and *Clostridioides difficile* infection (CDI) were evaluated per 1,000 patient-days. Resistant microorganisms included newly identified carbapenem-resistant *Enterobacterales* (CRE), carbapenem-resistant *Pseudomonas aeruginosa* (CRPA), two-drug-resistant *P. aeruginosa*, multidrug-resistant *P. aeruginosa* (MDRP), MRSA, and third-generation cephalosporin-resistant *Escherichia coli* and *Klebsiella pneumoniae*. Duplicate detections of the same organism from the same patient within a given month were not double counted, whereas multiple organisms from the same patient were counted separately by species. Outpatient specimens were excluded. “New” was defined as cases in which the target organism was newly detected during the study month without detection of the same organism in the preceding 90 days. “Hospital-acquired” was defined as new detections from specimens collected on day 4 of hospitalization or later. CDI was diagnosed by stool immunochromatographic testing when glutamate dehydrogenase (GDH) and toxin results were positive. If the GDH result was positive but the toxin result was negative, CDI was confirmed by stool genetic testing for toxin; if negative, testing was terminated. CRE was defined as isolates resistant to meropenem (MEPM) or resistant to imipenem and cefmetazole, according to CLSI M100 (2012). Data on resistant microorganisms were generally obtained from the Japan Surveillance for Infection Prevention and Healthcare Epidemiology (J-SIPHE) system. However, CRE data were handled separately because the CRE definition used by JANIS was revised following changes to the reporting criteria in the Japanese National Institute of Infectious Diseases surveillance program. The revised definition was introduced on October 1, 2025, and the J-SIPHE system consequently applied the former criteria to data collected between January 2013 and December 2024 and the updated criteria to data from January 2025 onward. Under the former criteria, isolates were classified as CRE if they met either of the following conditions: (1) meropenem (MEPM) susceptibility was reported as “I,” “IR,” or “R”; or (2) imipenem susceptibility was reported as “I,” “IR,” or “R,” and cefmetazole susceptibility was reported as “R.” Under the updated criteria, isolates are classified as CRE if MEPM susceptibility is reported as “I,” “IR,” or “R.” Because the definition changed during the study period, CRE data were not obtained from the J-SIPHE system. Instead, CRE data based on the former criteria were obtained from the microbiology laboratory and aggregated for analysis. CRPA was defined as *P. aeruginosa* resistant to imipenem or MEPM, according to CLSI M100 (2012). Two-drug–resistant *P. aeruginosa* was defined as resistance to any two of the following classes according to broth microdilution testing: carbapenems, fluoroquinolones, and amikacin (including isolates intermediate or resistant to amikacin), according to CLSI M100 (2012). MDRP was defined by meeting all three of the following criteria: (1) imipenem minimum inhibitory concentration (MIC) ≥16 μg/mL or inhibition zone ≤13 mm on disk diffusion, (2) amikacin MIC ≥32 μg/mL or inhibition zone ≤14 mm, and (3) ciprofloxacin MIC ≥4 μg/mL or inhibition zone ≤15 mm. Resistance to third-generation cephalosporins was defined as resistance (R) to cefotaxime, ceftriaxone, or ceftazidime, according to CLSI M100 (2012).

Cost of antimicrobial agents

To evaluate the economic impact of the tele-stewardship program, the cost of antimicrobial purchases per patient-day was calculated. The adjusted purchase costs were calculated on the basis of the actual purchase costs (considering the cost of switching from branded to generic products and changes in drug prices) and prices of branded agents. The exchange rate was set at $1 = 150 Japanese yen as of March 2025.

Culture submission rate

The culture submission rate was defined as the number of submitted specimens divided by inpatient-days, multiplied by 1,000.

All-cause in-hospital mortality rate and length of hospital stay

To assess the clinical impact of tele-stewardship program implementation, all-cause in-hospital mortality within 30 days and length of hospital stay were analyzed.

Underlying conditions

Underlying conditions of the consulted patients were categorized as follows: (1) solid tumors, (2) hematologic malignancies, (3) solid and hematologic malignancies, and (4) non-malignant conditions.

Response preparation time

Response preparation time was defined as the interval between the responder’s login time and the time the response was submitted. When multiple consultations were handled within a single login session, only the time to the first response submission was counted. Entries consisting solely of acknowledgments (e.g., “thank you”) or evaluation comments were excluded from the analysis.

Response time

Response time was defined for each message as the interval between the time the consultation message was posted by the requester and the time the responder provided the first response.

**References**

1. Itoh N, Akazawa-Kai N, Yamaguchi M, Kawabata T. Efficiency of a long-term infectious diseases consultation and antimicrobial stewardship program at a Japanese cancer center: an interrupted time-series analysis. *Open Forum Infect Dis* 2024;11:ofae678.

2. Itoh N, Akazawa N, Kanawaku E, et al. Effects of infectious disease consultation and antimicrobial stewardship program at a Japanese cancer center: an interrupted time-series analysis. *PLoS One* 2022;17:e0263095.
