# Supplementary material for: Impact of an eConsult-based antimicrobial stewardship program using the ExpertTWIN system: a Bayesian structural time-series analysis
Source: Antimicrob Steward Healthc Epidemiol. 2026 Sep 7;6(1):e255. doi: 10.1017/ash.2026.10851 (PMC13760392; doi:10.1017/ash.2026.10851)
Supplement: Supplementary file 3 [file S2732494X26108511sup003.docx]

**Supplementary Tables**

**Supplementary Table 1.** Restriction of antibiotic supply during the study period

| Antimicrobial agent | Category | Restriction start date (Y/M/D) | Restriction end date (Y/M/D) | Restriction type |
| --- | --- | --- | --- | --- |
| AMPC | Tablet | 2023/9/14 | Ongoing | Usage-restricted^a^ |
| AMPC/CVA (Augmentin) | Tablet | 2023/8/22 | Ongoing | Order-stopped^b^ |
| AMPC/CVA (Clavamox) | Tablet | 2023/6/5 | 2025/2/25 | Order-stopped^b^ |
| SBTPC | Tablet | 2023/6/19 | Ongoing | Usage-restricted^a^ |
| SBT/ABPC | Injection | 2023/3/7 | 2024/4/1 | Usage-restricted^a^ |
| CEX | Tablet | 2023/9/29 | 2024/7/24 | Usage-restricted^a^ |
| CEX | Tablet | 2024/7/25 | Ongoing | Order-stopped^b^ |
| CTM | Injection | 2023/11/29 | 2025/2/12 | Order-stopped^b^ |
| CCL | Tablet | 2023/10/17 | 2024/6/19 | Usage-restricted^a^ |
| CCL | Tablet | 2024/6/20 | 2026/1/5 | Order-stopped^b^ |
| CCL | Tablet | 2026/1/6 | Ongoing | Usage-restricted^a^ |
| CMZ | Injection | 2022/7/29 | Ongoing | Usage-restricted^a^ |
| CPZ/SBT | Injection | 2023/4/27 | 2024/9/24 | Order-stopped^b^ |
| FMOX | Injection | 2022/8/9 | 2024/12/17 | Usage-restricted^a^ |
| DRPM | Injection | 2022/8/23 | Ongoing | Usage-restricted^a^ |
| MEPM | Injection | 2022/8/22 | Ongoing | Usage-restricted^a^ |
| AZM | Tablet | 2023/6/19 | 2025/6/11 | Usage-restricted^a^ |
| EM | Tablet | 2024/4/5 | 2025/5/28 | Usage-restricted^a^ |
| MINO | Injection | 2022/11/7 | 2025/1/5 | Usage-restricted^a^ |
| MINO | Injection | 2025/1/6 | 2025/9/4 | Order-stopped^b^ |
| ST | Injection | 2025/8/1 | Ongoing | Usage-restricted^a^ |

Abbreviations: AMPC, amoxicillin; AMPC/CVA, amoxicillin/clavulanic acid; SBTPC, sultamicillin tosilate hydrate; SBT/ABPC, sulbactam/ampicillin; CEX, cephalexin; CTM, cefotiam; CCL, cefaclor; CMZ, cefmetazole; CPZ/SBT, cefoperazone/sulbactam; FMOX, flomoxef; DRPM, doripenem; MEPM, meropenem; AZM, azithromycin; EM, erythromycin; MINO, minocycline; ST, sulfamethoxazole/trimethoprim; Y/M/D, year/month/day.

^a^Usage-restricted refers to situations in which no restrictions are implemented within the ordering system, although prescribers are advised to modify prescriptions on a case-by-case basis due to stock shortages.

^b^Order-stopped refers to the complete suspension of prescription orders, with use permitted only for specific patients under special circumstances.

**Supplementary Table 2.** Content and acceptance rate of feedback by the telestewardship program with regard to specific broad-spectrum antimicrobial usage

| Date (Y/M) | Number of evaluations (n) | Suggestion (n) | Accepted (n, %)^a^ | Category | | | | | | | | |
| --- | --- | --- | --- | --- | --- | --- | --- | --- | --- | --- | --- | --- |
|  |  |  |  | **A** | **B** | **C** | **D** | **E** | **F** | **H** | **I** | **T** |
| 2025/2 | 8 | 14 | 9 (64.3) | 0 | 0 | 0 | 3 | 0 | 0 | 5 | 0 | 0 |
| 2025/3 | 10 | 24 | 9 (37.5) | 0 | 0 | 1 | 3 | 0 | 0 | 6 | 0 | 0 |
| 2025/4 | 11 | 14 | 6 (42.9) | 0 | 0 | 0 | 5 | 0 | 0 | 6 | 0 | 0 |
| 2025/5 | 12 | 14 | 7 (50.0) | 0 | 1 | 0 | 7 | 0 | 0 | 4 | 0 | 0 |
| 2025/6 | 18 | 25 | 20 (80.0) | 0 | 0 | 1 | 14 | 0 | 0 | 3 | 0 | 0 |
| 2025/7 | 15 | 22 | 16 (72.7) | 0 | 0 | 0 | 8 | 1 | 0 | 6 | 0 | 0 |
| 2025/8 | 11 | 13 | 8 (61.5) | 0 | 0 | 0 | 6 | 0 | 0 | 5 | 0 | 0 |
| 2025/9 | 14 | 17 | 12 (70.6) | 0 | 0 | 0 | 5 | 1 | 0 | 8 | 0 | 0 |
| 2025/10 | 15 | 23 | 17 (73.9) | 0 | 0 | 0 | 7 | 0 | 0 | 8 | 0 | 0 |
| 2025/11 | 10 | 17 | 13 (76.5) | 0 | 0 | 0 | 4 | 1 | 0 | 5 | 0 | 0 |
| 2025/12 | 7 | 11 | 5 (45.5) | 0 | 1 | 1 | 0 | 0 | 0 | 5 | 0 | 0 |
| 2026/1 | 14 | 20 | 13 (65.0) | 0 | 0 | 1 | 7 | 0 | 0 | 6 | 0 | 0 |

Abbreviations: A, appropriate; B, better choice; C, culture; D, de-escalation; E, escalation; F, fitting dose; H, halt; I, indication document; T, time out; Y/M, year/month.

^a^Acceptance rate is the sum of accepted suggestions divided by the total number of suggestions.

**Supplementary Table 3.** Sensitivity analyses of the primary outcome: carbapenem DOTs per 100 patient-days per month.

| Components in BSTS model | Average  effect | 95% CrI | PP of the hypothesized　effect direction (%) | ESS | R-hat |
| --- | --- | --- | --- | --- | --- |
| Local level | −1.4 | (−1.7, −1.0) | 99.9% | 31996.0 | 1.00 |
| Local level + seasonality | −1.4 | (−1.5, −1.2) | 99.9% | 33678.9 | 1.00 |
| Local linear trend | −2.9 | (−7.3, −0.2) | 98.1% | 1198.7 | 1.00 |
| Poisson model (only local level component) | −2.2 | (−4.2, −0.8) | 99.9% | 30042.0 | 1.00 |

Abbreviations: BSTS, Bayesian structural time-series; CrI, credible interval; DOTs, days of therapy; ESS, effective sample size; MCMC, Markov chain Monte Carlo; PP, Posterior probability; R-hat, potential scale reduction factor.

**Supplementary Table 4.** Posterior Estimates and MCMC Convergence Diagnostics for All Outcomes Analyzed Using Bayesian Structural Time-Series Models.

|  | Average  effect | 95% CrI | PP of the hypothesized　effect direction (%) | ESS | R-hat |
| --- | --- | --- | --- | --- | --- |
| Use of antimicrobial agents | | | | | |
| DOTs for carbapenems^a^ per 100 patient-days per month | −1.4 | (−1.7, −1.0) | 99.9% | 31996.0 | 1.00 |
| DOTs for antipseudomonal agents^b^ per 100 patient-days per month | 0.3 | (−0.1, 0.7) | 93.3% | 31745.8 | 1.00 |
| DOTs for narrow-spectrum antibiotics^c^ per 100 patient-days per month | 0.6 | (−0.1, 1.4) | 94.8% | 32820.2 | 1.00 |
| DOTs for anti-MRSA antimicrobial agents^d^ per 100 patient-days per month | −0.2 | (−0.6, 0.1) | 92.1% | 30735.7 | 1.00 |
| DOTs for all intravenous antimicrobial agents per 100 patient-days per month | −0.7 | (−2.3, 0.9) | 81.9% | 31092.6 | 1.00 |
| DOTs for all oral antimicrobial agents per 100 patient-days per month | −1.2 | (−3.6, 1.3) | 84.0% | 31060.4 | 1.00 |
| DOTs for total intravenous and oral antimicrobial agents per 100 patient-days per month | −1.9 | (−5.3, 1.6) | 86.7% | 31026.2 | 1.00 |
| Incidence of antimicrobial-resistant microorganisms | | | | | |
| CRE per 1,000 patient-days per month^1^ | – | – | – | – | – |
| Third-generation cephalosporin-resistant *Escherichia coli* per 1,000 patient-days per month | 0.20 | (0.06, 0.33) | 99.7% | 31692.7 | 1.00 |
| Third-generation cephalosporin-resistant *Klebsiella pneumoniae* per 1,000 patient-days per month^1^ | – | – | – | – | – |
| CDI per 1,000 patient-days per month | −0.01 | (−0.10, 0.08) | 61.7% | 30764.8 | 1.00 |
| CRPA per 1,000 patient-days per month | −0.04 | (−0.09, 0.01) | 93.7% | 30853.9 | 1.00 |
| Two-drug-resistant *Pseudomonas aeruginosa* per 1,000 patient-days per month | −0.04 | (−0.10, 0.02) | 90.3% | 30920.5 | 1.00 |
| MDRP per 1,000 patient-days per month^1^ | – | – | – | – | – |
| MRSA per 1,000 patient-days per month | −0.14 | (−0.40, 0.12) | 86.8% | 31439.5 | 1.00 |
| Laboratory metric | | | | | |
| Culture submission rate per 1,000 patient-days per month | 3.9 | (−2.4, 9.8) | 89.3% | 31475.8 | 1.00 |
| Patient outcome | | | | | |
| All-cause in-hospital mortality | −0.09 | (−0.47, 0.29) | 67.7% | 31996.0 | 1.00 |
| Average length of hospital stay | −0.12 | (−0.52, 0.26) | 73.3% | 31736.6 | 1.00 |

Abbreviations: BSTS, Bayesian structural time-series; CDI, *Clostridioides difficile infection*; CRE, carbapenem-resistant Enterobacterales; CrI, credible interval; CRPA, carbapenem-resistant *Pseudomonas aeruginosa*; DOTs, days of therapy; ESS, effective sample size; MCMC, Markov chain Monte Carlo; MDRP, multidrug-resistant *Pseudomonas aeruginosa*; MRSA, methicillin-resistant *Staphylococcus aureus*; PP, Posterior probability; R-hat, potential scale reduction factor.

^a^Imipenem/cilastatin, meropenem, and doripenem.

^b^Cefepime, cefozopran, piperacillin/tazobactam, and ceftolozane/tazobactam.

^c^Penicillin G, ampicillin, ampicillin/sulbactam, cefazolin, cefmetazole, and flomoxef.

^d^Vancomycin, teicoplanin, daptomycin, linezolid and tedizolid.

^1^Because the pre-intervention data were zero, the causal impact analysis could not be applied.
